# New Insights into Human Nostril Microbiome from the Expanded Human Oral Microbiome Database (eHOMD): a Resource for the Microbiome of the Human Aerodigestive Tract

**DOI:** 10.1128/mSystems.00187-18

**Published:** 2018-12-04

**Authors:** Isabel F. Escapa, Tsute Chen, Yanmei Huang, Prasad Gajare, Floyd E. Dewhirst, Katherine P. Lemon

**Affiliations:** a The Forsyth Institute (Microbiology), Cambridge, Massachusetts, USA; b Department of Oral Medicine, Infection & Immunity, Harvard School of Dental Medicine, Boston, Massachusetts, USA; c Division of Infectious Diseases, Boston Children’s Hospital, Harvard Medical School, Boston, Massachusetts, USA; Qingdao Institute of Bioenergy and Bioprocess Technology, Chinese Academy of Sciences

**Keywords:** 16S, *Corynebacterium*, *Dolosigranulum*, *Lawsonella*, nares, *Staphylococcus*, microbiota, nasal, respiratory tract, sinus

## Abstract

The expanded Human Oral Microbiome Database (eHOMD) is a comprehensive microbiome database for sites along the human aerodigestive tract that revealed new insights into the nostril microbiome. The eHOMD provides well-curated 16S rRNA gene reference sequences linked to available genomes and enables assignment of species-level taxonomy to most next-generation sequences derived from diverse aerodigestive tract sites, including the nasal passages, sinuses, throat, esophagus, and mouth. Using minimum entropy decomposition coupled with the RDP Classifier and our eHOMD V1-V3 training set, we reanalyzed 16S rRNA V1-V3 sequences from the nostrils of 210 Human Microbiome Project participants at the species level, revealing four key insights. First, we discovered that Lawsonella clevelandensis, a recently named bacterium, and *Neisseriaceae* [G-1] HMT-174, a previously unrecognized bacterium, are common in adult nostrils. Second, just 19 species accounted for 90% of the total sequences from all participants. Third, 1 of these 19 species belonged to a currently uncultivated genus. Fourth, for 94% of the participants, 2 to 10 species constituted 90% of their sequences, indicating that the nostril microbiome may be represented by limited consortia. These insights highlight the strengths of the nostril microbiome as a model system for studying interspecies interactions and microbiome function. Also, in this cohort, three common nasal species (Dolosigranulum pigrum and two *Corynebacterium* species) showed positive differential abundance when the pathobiont Staphylococcus aureus was absent, generating hypotheses regarding colonization resistance. By facilitating species-level taxonomic assignment to microbes from the human aerodigestive tract, the eHOMD is a vital resource enhancing clinical relevance of microbiome studies.

**IMPORTANCE** The eHOMD (http://www.ehomd.org) is a valuable resource for researchers, from basic to clinical, who study the microbiomes and the individual microbes in body sites in the human aerodigestive tract, which includes the nasal passages, sinuses, throat, esophagus, and mouth, and the lower respiratory tract, in health and disease. The eHOMD is an actively curated, web-based, open-access resource. eHOMD provides the following: (i) species-level taxonomy based on grouping 16S rRNA gene sequences at 98.5% identity, (ii) a systematic naming scheme for unnamed and/or uncultivated microbial taxa, (iii) reference genomes to facilitate metagenomic, metatranscriptomic, and proteomic studies and (iv) convenient cross-links to other databases (e.g., PubMed and Entrez). By facilitating the assignment of species names to sequences, the eHOMD is a vital resource for enhancing the clinical relevance of 16S rRNA gene-based microbiome studies, as well as metagenomic studies.

## Abstract (precis)

The eHOMD (http://www.ehomd.org) is a valuable resource for researchers, from basic to clinical, who study the microbiomes and the individual microbes in body sites in the human aerodigestive tract, which includes the nasal passages, sinuses, throat, esophagus, and mouth, and the lower respiratory tract, in health and disease. The eHOMD is an actively curated, web-based, open-access resource. eHOMD provides the following: (i) species-level taxonomy based on grouping 16S rRNA gene sequences at 98.5% identity, (ii) a systematic naming scheme for unnamed and/or uncultivated microbial taxa, (iii) reference genomes to facilitate metagenomic, metatranscriptomic, and proteomic studies and (iv) convenient cross-links to other databases (e.g., PubMed and Entrez). By facilitating the assignment of species names to sequences, the eHOMD is a vital resource for enhancing the clinical relevance of 16S rRNA gene-based microbiome studies, as well as metagenomic studies.

## INTRODUCTION

The human aerodigestive tract, which includes the oral cavity, pharynx, esophagus, nasal passages, and sinuses, commonly harbors both harmless and pathogenic bacterial species of the same genus. Therefore, optimizing the clinical relevance of microbiome studies for body sites within the aerodigestive tract requires sequence identification at the species level or at least the subgenus level. Understanding the composition and function of the microbiome of the aerodigestive tract is important for understanding human health and disease since aerodigestive tract sites are often colonized by common bacterial pathogens and are associated with prevalent diseases characterized by dysbiosis.

The reductions in the cost of next-generation DNA sequencing (NGS) combined with the increasing ease of determining bacterial community composition using short NGS-generated 16S rRNA gene fragments now make this a practical approach for large-scale molecular epidemiological, clinical, and translational studies (1). Optimal clinical relevance of such studies requires at least species-level identification (2); however, to date, 16S rRNA gene tag studies of the human microbiome are overwhelmingly limited to genus-level resolution. For example, many studies of nasal microbiota fail to distinguish medically important pathogens, e.g., Staphylococcus aureus, from generally harmless members of the same genus, e.g., Staphylococcus epidermidis. For many bacterial taxa, newer computational methods, e.g., Minimum Entropy Decomposition (MED), an unsupervised form of oligotyping (3), and DADA2 (4), parse NGS-generated short 16S rRNA gene sequences to species-level, sometimes strain-level, resolution. However, to achieve species-level taxonomy assignment for the resulting oligotypes/amplicon sequence variants, these methods must be used in conjunction with a high-resolution 16S rRNA gene taxonomic database and a classifying algorithm. Similarly, metagenomic sequencing provides species-level, and often strain-level, resolution when coupled with a reference database that includes genomes from multiple strains for each species. For the mouth, the Human Oral Microbiome Database (HOMD) (5, 6) has enabled analysis/reanalysis of oral 16S rRNA gene short-fragment data sets with these new computational tools, revealing microbe-microbe and host-microbe species-level relationships (7–9), and has been a resource for easy access to genomes from which to build reference sets for metagenomic and metatranscriptomic studies. In the expanded Human Oral Microbiome Database (eHOMD), we have considerably increased the number of genomes linked to aerodigestive tract taxa. Thus, the eHOMD (http://www.ehomd.org) is a comprehensive web-based resource enabling the broad community of researchers studying the nasal passages, sinuses, throat, esophagus, and mouth to leverage newer high-resolution approaches to study the microbiome of aerodigestive tract body sites in human in health and disease. The eHOMD should also serve as an effective resource for lower respiratory tract (LRT) microbiome studies based on the breadth of taxa included and the fact that many LRT microbes are found in the mouth, pharynx, and nasal passages (10).

The eHOMD also facilitates rapid comparison of 16S rRNA gene sequences from studies worldwide by providing a systematic provisional naming scheme for unnamed taxa identified through sequencing (6). Each high-resolution taxon in eHOMD, as defined by 98.5% sequence identity across close-to-full-length 16S rRNA gene sequences, is assigned a unique human microbial taxon (HMT) number that can be used to search and retrieve that sequence-based taxon from any data set or database. This stable provisional taxonomic scheme for unnamed and uncultivated taxa is one of the strengths of eHOMD, since taxon numbers stay the same even when names change.

In the first section of Results and Discussion, “The eHOMD is a resource for microbiome research on the human upper digestive and respiratory tracts,” we describe the process of generating the eHOMDv15.1 (http://www.ehomd.org) and its utility using both 16S rRNA gene clone library and short-read data sets. In the second section, “The eHOMD revealed previously unknown properties of the human nasal microbiome,” we report new discoveries about the nostril microbiome based on analysis using the eHOMD.

## RESULTS AND DISCUSSION

### The eHOMD is a resource for microbiome research on the human upper digestive and respiratory tracts.

As described below, the eHOMD (http://www.ehomd.org) is a comprehensive, actively curated, web-based resource open to the entire scientific community that classifies 16S rRNA gene sequences at a high resolution (98.5% sequence identity). Further, the eHOMD provides a systematic provisional naming scheme for as-yet unnamed/uncultivated taxa and a resource for easily searching available genomes for included taxa, thereby facilitating the identification of aerodigestive and lower respiratory tract bacteria and providing phylogenetic (http://ehomd.org/index.php?name=HOMD&show_tree=_), genomic, phenotypic, clinical, and bibliographic information for these microbes.

**FIG 1:**
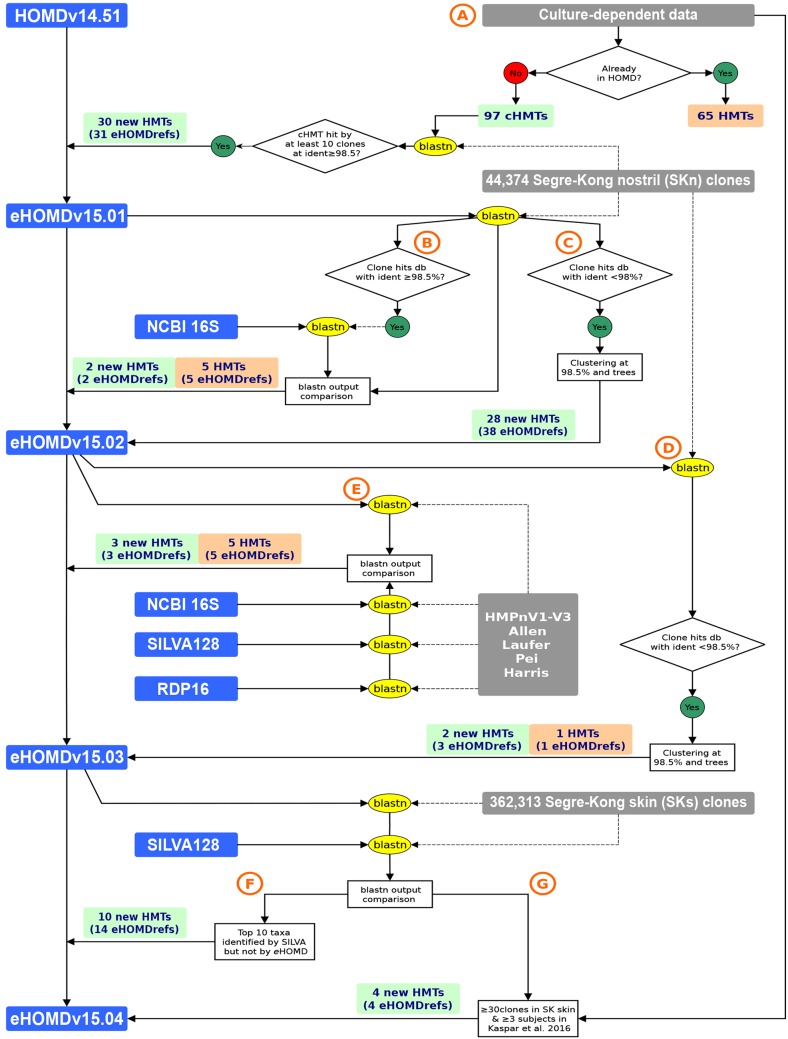
The process for identifying human microbial taxa (HMTs) from the aerodigestive tract to generate the eHOMD. Schematic of the approach used to identify taxa that were added as human microbial taxa (HMT) to generate the eHOMDv15.04. The colored boxes indicate databases (blue), data sets (gray), newly added HMTs (green), and newly added eHOMDrefs for the present HMTs (orange). The performance of blastn searches is indicated by yellow ovals and performance of other tasks is indicated in white rectangles. HMT replaces the old HOMD taxonomy prefix HOT (human oral taxon). (A) Process for generating the provisional eHOMDv15.01 by adding bacterial species from culture-dependent studies. (B and C) Process for generating the provisional eHOMDv15.02 by identifying additional HMTs from a data set of 16S rRNA gene clones from human nostrils. (D and E) Process for generating the provisional eHOMDv15.03 by identifying additional candidate taxa from culture-independent studies of aerodigestive tract microbiomes. (F and G) Process for generating the provisional eHOMDv15.04 by identifying additional candidate taxa from a data set of 16S rRNA gene clones from human skin. Please see Materials and Methods for detailed description of the processes depicted in panels A to G. Abbreviations: NCBI 16S, NCBI 16 Microbial database; eHOMDref, eHOMD reference sequence; db, database; ident, identity. Data sets included SKns (11–16), Allen et al. (22), Laufer et al. (21), Pei et al. (25, 26), Harris et al. (27), and Kaspar et al. (19).

**(i) The eHOMD captures the breadth of diversity of the human nostril microbiome.** Here we describe the generation of eHOMDv15.1, which performed as well or better than four other commonly used 16S rRNA gene databases (SILVA128, RDP16, NCBI 16S, and Greengenes GOLD) in assigning species-level taxonomy via blastn to sequences in a data set of nostril-derived 16S rRNA gene clones (Table 1) and short-read fragments (Table 2). Species-level taxonomy assignment was defined as 98.5% identity with 98% coverage via blastn (based on analysis shown in Fig. S1 in the supplemental material). An initial analysis showed that the oral-microbiome-focused HOMDv14.5 enabled species-level taxonomic assignment of only 50.2% of the 44,374 16S rRNA gene clones from nostril (anterior nares) samples generated by Julie Segre, Heidi Kong, and colleagues (henceforth the SKn data set) (Table 1) (11–16). To expand HOMD to be a resource for the microbiomes of the entire human aerodigestive tract, we started with the addition of nose- and sinus-associated bacterial species. As illustrated in Fig. 1 and described in detail in Materials and Methods, we compiled a list of candidate nasal and sinus species gleaned from culture-dependent studies (17–19) plus anaerobes cultivated from cystic fibrosis sputum samples (20) (see Table S1A in the supplemental material). To assess which of these candidate species are most likely to be common members of the nasal microbiome, we used blastn to identify those taxa present in the SKn data set. We then added one or two representative close-to-full-length 16S rRNA gene sequences (eHOMDrefs) for each of these taxa to a provisional expanded database (Fig. 1A). Using blastn, we assayed how well this provisional eHOMDv15.01 captured clones in the SKn data set (Table S1B). Examination of sequences in the SKn data set that were not identified resulted in further addition of new HMTs, generating the provisional eHOMDv15.02 (Fig. 1B and C). Next, we evaluated how well eHOMDv15.02 served to identify sequences in the SKn clone data set using blastn (Fig. 1D). To evaluate its performance for other data sets compared to other databases, we took an iterative approach using blastn to evaluate the performance of eHOMDv15.02 against a set of three V1-V2 or V1-V3 16S rRNA gene short-read data sets (21–24) and two close-to-full-length 16S rRNA gene clone data sets from the aerodigestive tracts of healthy children and adults and those with a disease (25–27) in comparison to three commonly used 16S rRNA gene databases: NCBI 16S Microbial (NCBI 16S) (28), RDP16 (29), and SILVA128 (30, 31) (Fig. 1E and Table S1C). (We dropped Greengenes GOLD [32] from these subsequent steps because it identified only 70% of the SKn clones in the initial analysis in Table 1.) These steps resulted in the generation of the provisional eHOMDv15.03. Further additions to include taxa that can be present on the skin of the nasal vestibule (nostril or nares samples) but that are more common at other skin sites resulted from using blastn to analyze the full Segre-Kong skin 16S rRNA gene clone data set, excluding nostrils, (the SKs data set) (11–16) against both eHOMDv15.03 and SILVA128 (Fig. 1F and G). On the basis of these results, we generated the eHOMDv15.1, which identified 95.1% of the 16S rRNA gene reads in the SKn data set, outperforming the three other commonly used 16S rRNA gene databases (Table 1). Importantly, examination of the 16S rRNA gene phylogenetic tree of all eHOMDrefs in eHOMDv15.1 demonstrated that this expansion maintained the previous distinctions among oral taxa with the exception of *Streptococcus thermophilus,* which is >99.6% similar to *Streptococcus salivarius* and *Streptococcus vestibularis* (see Data S1A in the supplemental material and the current version of the phylogenetic tree at http://www.ehomd.org/ftp/HOMD_phylogeny/current). Each step in this process improved eHOMD with respect to identification of clones from the SKn data set, establishing eHOMD as a resource for the human nasal microbiome (Fig. 1 and Table S1B).

**TABLE 1:** The eHOMD outperforms comparable databases for species-level taxonomic assignment to 16S rRNA reads from nostril samples (SKn data set)

Database	No. of reads identified^*a*^	% reads identified^*a*^
HOMDv14.5	22,274	50.2
eHOMDv15.1	42,197	95.1
SILVA128	40,597	91.5
RDP16	38,815	87.5
NCBI 16S	38,337	86.4
Greengenes GOLD	31,195	70.3

a Reads identified via blastn search at 98.5% identity and 98% coverage.

**TABLE 2:** Performance of eHOMD and comparable databases for species-level taxonomic assignment to 16S rRNA gene data sets from sites throughout the human aerodigestive tract

Data set	16S region	16S primers	Sequencing technique	Sample type	No. of samples	No. of reads analyzed	Database	No. of reads identified^*a*^	% reads identified^*a*^
Laufer- Pettigrew (21)	V1-V2	27F, 338R	Roche/454	Nostril swab	108 children (108 samples)	120,274	eHOMDv15.1	96,233	80.0
							SILVA128	97,233	80.8
							RDP16	97,464	**81.0**
							NCBI 16S	87,082	72.4

Allen- Sale (22)	V1-V3	27F, 534R	454-FLX	Nasal lavage fluid	10 adults (97 samples)	75,310	eHOMDv15.1	68,594	91.1
							SILVA128	69,082	**91.7**
							RDP16	65,028	86.4
							NCBI 16S	63,892	84.8

Pei-Blaser (25, 26)	CL^*b*^	318F, 1519R, 8F, 1513R	CL	Esophageal biopsies	4 adults (10 libraries each)	7,414	eHOMDv15.1	7,276	**98.1**
							SILVA128	7,019	94.7
							RDP16	6,847	92.4
							NCBI 16S	6,686	90.2

Harris-Pace (27)	CL	27F, 907R	CL	Brochial alveola lavage fluid	57 children (50 libraries CF and 19 control)^*c*^	3,203	eHOMDv15.1	2,684	**83.8**
							SILVA128	2,633	82.2
							RDP16	2,500	78.1
							NCBI 16S	2,427	75.8

HMPnV1-V3 (23, 24)	V1-V3	27F, 534R	Roche/454	Nostril swab	227 adults (363 samples)^*d*^	2,338,563	eHOMDv15.1	2,133,083	**91.2**
							SILVA128	2,035,882	87.1
							RDP16	1,965,611	84.1
							NCBI 16S	1,932,732	82.6

van der Gast- Bruce (40)	CL	7F, 1510R	CL	Expectorated sputa	14 adults (CF)	2,137	eHOMDv15.1	2,123	**99.3**
							SILVA128	2,084	97.5
							RDP16	2,057	96.3
							NCBI 16S	2,045	95.7

Flanagan- Bristow (38)	CL	27F, 1492R	CL	Endotracheal tube aspirate	6 adults, 1 child (2 to 5 samples each)	3,278	eHOMDv15.1	3,193	97.4
							SILVA128	3,199	**97.6**
							RDP16	3,193	97.4
							NCBI 16S	3,186	97.2

Perkins- Angenent (39)	CL	8F, 1391R	CL	Extubated endotracheal tube	8 adults	1,263	eHOMDv15.1	1,008	**79.8**
							SILVA128	1,000	79.2
							RDP16	916	72.5
							NCBI 16S	832	65.9

a Reads identified via blastn search at 98.5% identity and 98% coverage.

b CL, clone library.

c CF, cystic fibrosis.

d See Text S1 in the supplemental material.

10.1128/mSystems.00187-18.2 DATA S1 Stable links to high-resolution visualizations at http://www.homd.org/ftp/publication_data/20180919/Supplemental_Figures/ of the phylogenetic trees referred to in this article (A to E). Download Data S1, DOCX file, 0.04 MB. Copyright © 2018 Escapa et al. 2018 Escapa et al. This content is distributed under the terms of the Creative Commons Attribution 4.0 International license.

10.1128/mSystems.00187-18.3 FIG S1 The percentage of 16S rRNA gene sequences identified via blastn search declines sharply at identity thresholds above 98.5% across the range of coverage tested. We analyzed blastn results of the SKn clone library data set (A), as an example of a full-length 16S rRNA gene data set, and the HMP nares V1-V3 16S rRNA data set (B), as an example of a short NGS-generated data set, against four different databases. The gray panels on top show the range of percent coverage used. The *x* axis represents the range of percent identity thresholds used. Each database is represented in a different color (see key). On the basis of these results, we chose to use a threshold of 98.5% identity and 98% coverage for blastn analysis. Download FIG S1, EPS file, 1.7 MB. Copyright © 2018 Escapa et al. 2018 Escapa et al. This content is distributed under the terms of the Creative Commons Attribution 4.0 International license.

10.1128/mSystems.00187-18.4 TABLE S1 The expanded eHOMDv15.1 was generated by identifying candidate taxa from culture-dependent studies (A), 16S rRNA gene clones from human nostrils (B), and skin- and culture-independent studies of aerodigestive tract microbiomes (C). Download Table S1, XLSX file, 0.02 MB. Copyright © 2018 Escapa et al. 2018 Escapa et al. This content is distributed under the terms of the Creative Commons Attribution 4.0 International license.

SILVA128 identified the next largest percentage of the SKn clones (91.5%) to the species level by blastn with our criteria (Table 1). Of the 44,373 clones in the SKn data set, a common set of 90.2% were captured at 98.5% identity and 98% coverage by both databases but with differential species-level assignment for 15.6% (6,237) (Table S2A). Another 1.3% were identified only by SILVA (Table S2B), and 4.9% were identified only by eHOMDv15.1 (Table S2C). Of the differentially named SKn clones, 45% belong to the genus *Corynebacterium*. Therefore, we generated a tree of all of the reference sequences for *Corynebacterium* species from both databases (Data S1B). This revealed that the *Corynebacterium jeikeium* SILVA-JVVY01000068.479.1974 reference sequence forms a clade with *C. propinquum* references from both databases, indicating a misannotation in SILVA128. This accounted for 34.4% (2,147) of the differentially named clones, which eHOMD correctly attributed to *C. propinquum* (Table S2A). Another 207 SKn clones grouped with *C. fastidiosum* SILVA-AJ439347.1.1513. eHOMDv15.1 lacks this species, so it incorrectly attributed 3.3% (207) to *C. accolens*. The bulk of the remaining differentially named *Corynebacterium* species also resulted from misannotation of reference sequences in SILVA128, e.g., SILVA-JWEP01000081.32.1536 as *C. urealyticum*, JVXO01000036.12.1509 as *C. aurimucosum,* and SILVA-HZ485462.10.1507 as *C. pseudogenitalium*, which is not a validly recognized species name (Data S1B). Recently, Edgar estimated an annotation error of ∼17% in SILVA128 (33). Since eHOMD taxa are represented by just one to six highly curated eHOMDrefs, we minimize the misannotation issues observed in larger databases. At the same time, our deep analysis of the phylogenetic space of each taxon allows eHOMD to identify a high percentage of reads in aerodigestive tract data sets. Having compared eHOMDv15.1 and SILVA128, we next benchmarked the performance of eHOMDv15.1 for assigning taxonomy to both other 16S rRNA gene clone libraries and against short-read 16S rRNA fragment data sets from the human aerodigestive tract (Table 2).

10.1128/mSystems.00187-18.5 TABLE S2 Comparison of the taxonomic assignment to the species level by blastn search of the SKn clones using eHOMDv15.1 versus SILVA128 revealed a subset of reads that were classified as captured at 98.5% identity and 98% coverage by both databases but had differential species-level assignment (A) or were identified only with SILVA (B) or only with eHOMDv15.1 (C). Download Table S2, XLSX file, 0.02 MB. Copyright © 2018 Escapa et al. 2018 Escapa et al. This content is distributed under the terms of the Creative Commons Attribution 4.0 International license.

**(ii) The 16S rRNA gene V1-V3 region provides superior taxonomic resolution for bacteria from the human aerodigestive tract compared to the V3-V4 region that is commonly used in microbiome studies.** The choice of variable region for NGS-based short-read 16S rRNA gene microbiome studies impacts what level of phylogenetic resolution is attainable. For example, for skin, V1-V3 sequencing results show high concordance with those from metagenomic sequencing (34). Similarly, to enable species-level distinctions within respiratory tract genera that include both common commensals and pathogens, V1-V3 is preferable for the nasal passages, sinuses, and nasopharynx (2, 35–37). We observed that in eHOMDv15.1, only 14 taxa have 100% identity across the V1-V3 region, whereas 63 taxa have 100% identity across the V3-V4 region (Table 3). The improved resolution with V1-V3 was even more striking at 99% identity, with 37 taxa indistinguishable using V1-V3 compared to 269 taxa indistinguishable using V3-V4. Table S3A to F shows the subsets of taxa collapsing into undifferentiated groups at each percent identity threshold for the V1-V3 and V3-V4 regions. This analysis provides clear evidence that V1-V3 sequencing is necessary to achieve maximal species-level resolution for 16S rRNA gene-based microbiome studies of the human oral and respiratory tracts, i.e., the aerodigestive tract. Therefore, we used 16S rRNA gene V1-V2 or V1-V3 short-read data sets to assess the performance of eHOMDv15.1 in Table 2.

**TABLE 3:** Number of species-level taxa in eHOMDv15.1 that are indistinguishable at various percent identity thresholds for 16S rRNA regions V1-V3 and V3-V4

% identity	No. of taxa that are indistinguishable	
	V1-V3	V3-V4
99	37	269
99.5	22	171
100	14	63

10.1128/mSystems.00187-18.6 TABLE S3 Subsets of taxa that collapsed into undifferentiated groups at each percent identity threshold (100%, 99.5%, and 99%) for the V1-V3 regions (A to C) and V3-V4 regions (D to F) of the 16S rRNA gene. Download Table S3, XLSX file, 0.04 MB. Copyright © 2018 Escapa et al. 2018 Escapa et al. This content is distributed under the terms of the Creative Commons Attribution 4.0 International license.

**(iii) The eHOMD is a resource for taxonomic assignment of 16S rRNA gene sequences from the entire human aerodigestive tract and the lower respiratory tract.** To assess its performance and the value for analysis of data sets from sites throughout the human aerodigestive tract, eHOMDv15.1 was compared with three commonly used 16S rRNA gene databases and consistently performed better than or comparable to these databases (Table 2). For these comparisons, we used blastn to assign taxonomy to three short-read (V1-V2 and V1-V3) and five approximately full-length clone library 16S rRNA gene data sets from the human aerodigestive tract that are publicly available (21–23, 25–27, 38–40). For short-read data sets, we focused on those covering all or part of the V1-V3 region of the 16S rRNA gene for the reasons discussed above. The chosen data sets include samples from children or adults who were healthy and/or had a disease. The samples in these data sets are from human nostril swabs (21, 23), nasal lavage fluid specimens (22), esophageal biopsy specimens (25, 26), extubated endotracheal tubes (39), endotracheal tube aspirate specimens (38), sputum specimens (40), and bronchoalveolar lavage (BAL) fluid specimens (27). Endotracheal tube sampling may represent both upper and lower respiratory tract microbes, and sputum may be contaminated by oral microbes, whereas BAL fluid contains microbes present in the lower respiratory tract. Therefore, these provide broad representation for bacterial microbiota of the human aerodigestive tract, as well as the human lower respiratory tract (Table 2). The composition of the bacterial microbiota from the nasal passages varies across the human life span (1), and eHOMD captures this variability. The performance of eHOMDv15.1 in Table 2 establishes it as a resource for microbiome studies of body sites within the human respiratory and upper digestive tracts.

The eHOMDv15.1 performed very well for nostril samples (Tables 1 and 2), which are a type of skin microbiome sample, since the nostrils open onto the skin-covered surface of the nasal vestibules. Because of this, we hypothesized that eHOMD might also perform well for other skin sites. To test this hypothesis, we used eHOMDv15.04 to perform blastn for taxonomic assignment of 16S rRNA gene reads from the complete set of clones from multiple nonnasal skin sites generated by Segre, Kong, and colleagues (SKs data set) (11–16). As shown in Table 4, eHOMDv15.04 performed very well for oily skin sites (alar crease, external auditory canal, back, glabella, manubrium, retroauricular crease, and occiput) and the nostrils (nares), identifying >88% of the clones, which was more than the other databases for six of these eight sites. Either SILVA128 or eHOMDv15.04 consistently identified the most clones for each skin site to the species level (98.5% identity and 98% coverage); the performance of eHOMDv15.04 was almost identical to the performance of eHOMDv15.1. In contrast, eHOMDv15.04 performed less well than SILVA128 for the majority of the moist skin sites (Table 4), e.g., the axillary vault (armpit). A review of the details of these results revealed that a further expansion comparable to what we did to go from a mouth-focused to an aerodigestive tract-focused database is necessary for eHOMD to include the full diversity of all skin sites.

**TABLE 4:**
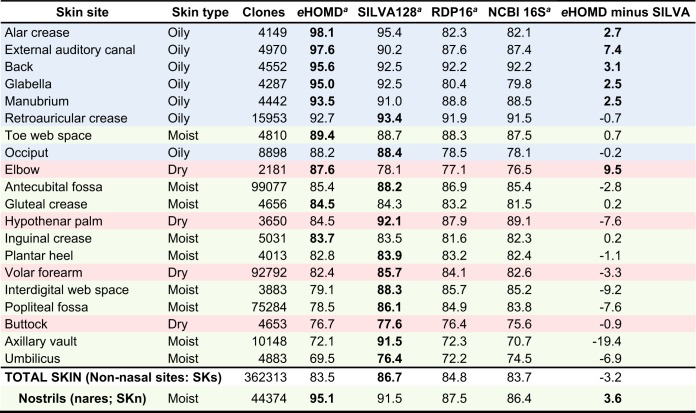
For nonnasal skin samples, the eHOMD performs best for species-level taxonomic assignment to 16S rRNA reads from oily skin sites (SKs data set)

a Reads identified via blastn search at 98.5% identity and 98% coverage. The skin type is indicated in color as follows: oily (blue), dry (red), and moist (green).

**(iv) The eHOMD is a resource for annotated genomes matched to HMTs for use in metagenomic and metatranscriptomic studies.** Well-curated and annotated reference genomes correctly named at the species level are a critical resource for mapping metagenomic and metatranscriptomic data to gene and functional information and for identifying species-level activity within the microbiome. There are currently >160,000 microbial genomic sequences deposited in GenBank; however, many of these genomes remain poorly annotated or have not yet been annotated or lack species-level taxonomy assignment, thus limiting the functional interpretation of metagenomic/metatranscriptomic studies to the genus level. Therefore, as an ongoing process, one goal of the eHOMD is to provide correctly named, curated, and annotated genomes for all HMTs. In generating eHOMDv15.1, we determined the species-level assignment for 117 genomes in GenBank that were previously identified only to the genus level and that matched 25 eHOMD taxa (Data S1C and S1D). For each of these genomes, the phylogenetic relationship to the assigned HMT was verified by both phylogenetic analysis using 16S rRNA gene sequences (Data S1C) and by phylogenomic analysis using a set of core proteins and PhyloPhlAn (41) (Data S1D). To date, 85% (475) of the cultivated taxa (and 62% of all taxa) included in eHOMD have at least one sequenced genome.

**(v) The eHOMD is a resource for species-level assignment to the outputs of high-resolution 16S rRNA gene analysis algorithms.** Algorithms, such as DADA2 and MED, permit high-resolution parsing of 16S rRNA gene short-read sequences (3, 4). Moreover, the RDP naive Bayesian Classifier is an effective tool for assigning taxonomy to 16S rRNA gene sequences, both full-length and short reads, when coupled with a robust, well-curated training set (42, 43). Together, these tools permit species-level analysis of short-read 16S rRNA gene data sets. Because the V1-V3 region is the most informative short-read fragment for most of the common bacteria of the aerodigestive tract, we generated a training set for the V1-V3 region of the 16S rRNA gene that includes all taxa represented in the eHOMD, which is described elsewhere. In our training set, we grouped taxa that were indistinguishable based on the sequence of their V1-V3 region together as supraspecies to preserve subgenus-level resolution, e.g., *Staphylococcus capitis_caprae.*

**(vi) Advantages and limitations of the eHOMD.** The eHOMD has advantages and limitations compared to other 16S rRNA gene databases, such as RDP, NCBI, SILVA, and Greengenes (28–32). Its primary distinction is that eHOMD is dedicated to providing taxonomic, genomic, bibliographic, and other information specifically for the approximately 800 microbial taxa found in the human aerodigestive tract (summarized in Table 5). Here, we highlight five advantages of eHOMD. First, the eHOMD is based on extensively curated 16S rRNA reference sets (eHOMDrefs) and a taxonomy that uses phylogenetic position in 16S rRNA-based trees rather than a taxon’s currently assigned, or misassigned, taxonomic name (6). For example, the genus “*Eubacteria*” in the phylum *Firmicutes* includes members that should be divided into multiple genera in seven different families (44). In eHOMD, members of the “*Eubacteria”* are placed in their phylogenetically appropriate family, e.g., *Peptostreptococcaceae*, rather than incorrectly into the family *Eubacteriaceae.* Appropriate taxonomy files are readily available from eHOMD for mothur (45) and other programs. Second, because eHOMD includes a provisional species-level naming scheme, sequences that can only be assigned genus-level taxonomy in other databases are resolved to the species level via an HMT number. This enhances the ability to identify and learn about taxa that currently lack full identification and naming. Importantly, the HMT number is stable, i.e., it stays constant even as a taxon is named or the name is changed. This facilitates tracking knowledge of a specific taxon over time and between different studies. Third, in eHOMD, for the 475 taxa with at least one sequenced genome, genomes can be viewed graphically in the dynamic JBrowse genome web viewer (46) or searched using blastn, blastp, blastx, tblastn, or tblastx. For taxa lacking accessible genomic sequences, the available 16S rRNA sequences are included. Many genomes of aerodigestive tract organisms are in the whole-genome shotgun contigs (wgs) section of NCBI and are visible by blast search only through wgs, provided that one knows the genome and can provide the BioProjectID or WGS Project ID. At eHOMD, one can readily compare dozens to more than a hundred genomes for some taxa to begin to understand the pangenome of aerodigestive tract microbes. Fourth, we have also compiled proteome sequence sets for genome-sequenced taxa, enabling proteomic and mass spectra searches on a data set limited to proteins from ∼2,000 relevant genomes. Fifth, for analysis of aerodigestive tract 16S rRNA gene data sets, eHOMD is a focused collection and, therefore, smaller in size. This results in increased computational efficiency compared to the other databases. eHOMD performed a blastn search of ten 16S rRNA gene full-length reads in 0.277 s, while the same analysis with the NCBI 16 database took 3.647 s and RDP and SILVA needed more than 1 min (see Text S1 in the supplemental material).

**TABLE 5:** Summary of eHOMD data at the phylum level^*a*^

Phylum	No. of taxa	No. of eHOMDrefs	No. of genomes
*Absconditabacteria* (SR1)	5	3	1
*Actinobacteria*	118	153	292
*Bacteroidetes*	125	179	133
*Chlamydiae*	1	1	5
*Chlorobi*	3	0	3
*Chloroflexi*	3	1	4
*Cyanobacteria*	1	2	1
*Euryarchaeota*	1	0	1
*Firmicutes*	266	341	581
*Fusobacteria*	37	46	60
*Gracilibacteria* (GN02)	5	3	2
*Proteobacteria*	141	174	393
*Saccharibacteria* (TM7)	19	16	7
*Spirochaetes*	50	64	35
*Synergistetes*	8	15	8
WPS-2	1	0	1

**Total**	**784**	**998**	**1,527**

a Data were compiled at the time of writing this paper; for updated summary and at different taxonomy levels, visit the eHOMD web site (http://www.homd.org/index.php?name=HOMD&taxonomy_level=1).

10.1128/mSystems.00187-18.1 TEXT S1 Supplemental methods. Download Text S1, DOCX file, 0.08 MB. Copyright © 2018 Escapa et al. 2018 Escapa et al. This content is distributed under the terms of the Creative Commons Attribution 4.0 International license.

In terms of limitations, the taxa included in the eHOMD, the 16S rRNA reference sequences and genomes, are not appropriate for samples from (i) human body sites outside the aerodigestive and respiratory tracts, (ii) nonhuman hosts, or (iii) the environment. In contrast, RDP (29), SILVA (30, 31), and Greengenes (32) are curated 16S rRNA databases that include taxa from all sources and environments. The NCBI 16S database is a curated set of sequences for named bacterial and archaeal species only (also known as RefSeqs) that is frequently updated (28). Finally, the NCBI nucleotide database (nr/nt) includes the largest set of 16S rRNA sequences available; however, the vast majority have no taxonomic attribution and are listed as simply “uncultured bacterium clone.” Thus, RDP, SILVA, NCBI, Greengenes, and other similar general databases have advantages for research on microbial communities outside the human respiratory and upper digestive tracts, whereas eHOMD is preferred for the microbiomes of the human upper digestive and respiratory tracts.

### The eHOMD revealed previously unknown properties of the human nasal microbiome.

To date, the human nasal microbiome has mostly been characterized at the genus level. For example, the Human Microbiome Project (HMP) characterized the bacterial community in the adult nostrils (nares) to the genus level using 16S rRNA sequences (23, 24). However, the human nasal passages can host a number of genera that include both common commensals and important bacterial pathogens, e.g., *Staphylococcus, Streptococcus, Haemophilus, Moraxella,* and *Neisseria* (reviewed in reference 1). Thus, species-level nasal microbiome studies are needed from both a clinical and ecological perspective. Therefore, to further our understanding of the adult nostril microbiome, we used MED (3), the RDP classifier (42), and our eHOMD V1-V3 training set to reanalyze a subset of the HMP nares V1-V3 16S rRNA data set consisting of one sample each from 210 adults (see Materials and Methods). Henceforth, we refer to this subset as the HMP nares V1-V3 data set. This resulted in species/supraspecies-level taxonomic assignment for 95% of the sequences and revealed new insights into the adult nostril microbiome, which are described below.

**FIG 2:**
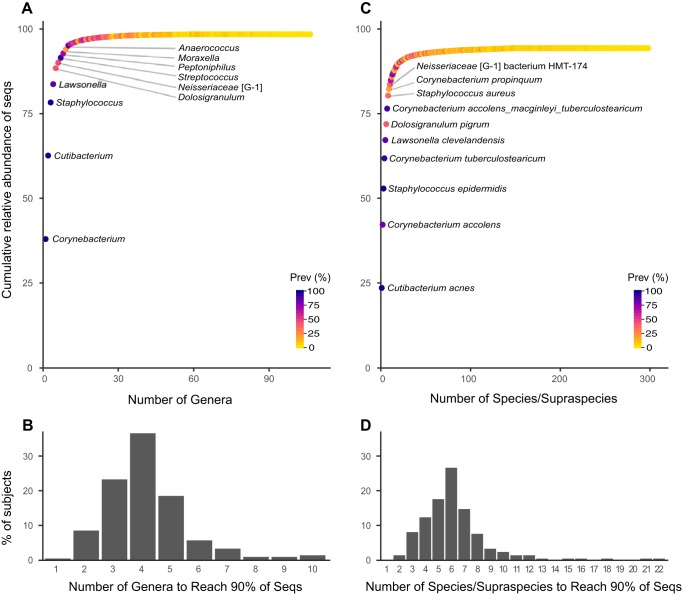
A small number of genera and species account for the majority of taxa in the HMP nares V1-V3 data set at both an overall level and individual level. (A and C) Taxa identified in the reanalysis of the HMP nostril V1-V3 data set graphed based on cumulative relative abundance of sequences at the genus level (A) and species/supraspecies level (C). The top 10 taxa are labeled. Prevalence (Prev) as a percentage is indicated by the color gradient. The genus *Cutibacterium* includes species formerly known as the cutaneous *Propionibacterium* species, e.g., Propionibacterium acnes (70). (B and D) The minimum number of taxa at the genus level (B) and species/supraspecies level (D) that accounted for 90% of the total sequences in each person’s sample based on a table of taxa ranked by cumulative abundance from greatest to least. Ten or fewer species/supraspecies accounted for 90% of the sequences in 94% of the 210 HMP participants in this reanalysis. The cumulative relative abundance of sequences does not reach 100% because 1.5% of the reads could not be assigned a genus and because 4.9% of the reads could not be assigned a species/supraspecies.

**(i) A small number of cultivated species account for the majority of the adult nostril microbiome.** Genus-level information from the HMP corroborates data from smaller cohorts showing that the nostril microbiome has a very uneven distribution both overall and per person (reviewed in reference 47). In our reanalysis, 10 genera accounted for 95% of the total reads from 210 adults (see Materials and Methods), with the remaining genera each present at very low relative abundance and prevalence (Fig. 2A and Table S4A). Moreover, for the majority of participants, five or fewer genera constituted 90% of the sequences in their sample (Fig. 2B). This uneven distribution characterized by the numeric dominance of a small number of taxa was even more striking at the species level (48). We found that the six most relatively abundant species made up 72% of the total sequences, and the top five each had a prevalence of ≥81% (Fig. 2C and Table S4B). Moreover, between 2 and 10 species accounted for 90% of the sequences in 94% of the participants (Fig. 2D). Also, just 19 species/supraspecies-level taxa constituted 90% of the total 16S rRNA gene sequences from all 210 participants (Table S4B), and one of these taxa belonged to an as-yet-uncultivated genus as described below. The implication of these findings is that *in vitro* consortia consisting of small numbers of cultivated species can effectively represent the natural nasal community, facilitating functional studies of the nostril microbiome.

10.1128/mSystems.00187-18.7 TABLE S4 Genus (A) and species/supraspecies (B) rank order abundance of sequences in the reanalysis of the HMP nares V1-V3 16S rRNA gene data set. Download Table S4, XLSX file, 0.03 MB. Copyright © 2018 Escapa et al. 2018 Escapa et al. This content is distributed under the terms of the Creative Commons Attribution 4.0 International license.

**(ii) Identification of two previously unrecognized common nasal bacterial taxa.** Reanalysis of both the HMP nares V1-V3 data set and the SKn 16S rRNA gene clone data set revealed that two previously unrecognized taxa are common in the nostril microbiome: Lawsonella clevelandensis and an unnamed *Neisseriaceae* [G-1] bacterium, to which we assigned the provisional name *Neisseriaceae* [G-1] bacterium HMT-174. These two bacterial taxa are discussed in further detail below.

**(iii) The human nasal passages are the primary habitat for a subset of bacterial species.** The topologically external surfaces of the human body are the primary habitat for a number of bacterial taxa, which are often present at both high relative abundance and high prevalence in the human microbiome. In generating eHOMDv15.1, we hypothesized that comparing the relative abundance of sequences identified to the species or supraspecies level in the SKn clones and the SKs clones (nonnasal skin sites) would permit putative identification of the primary body site habitat for a subset of nostril-associated bacteria. On the basis of criteria described in Materials and Methods, we putatively identified 13 species as having the nostrils and 1 species as having skin as their primary habitat (Table S5). Online at http://ehomd.org/index.php?name=HOMD, the primary body site for each taxon is denoted as oral, nasal, skin, vaginal, or unassigned. Definitive identification of the primary habitat of all human-associated bacteria will require species-level identification of bacteria at each distinct habitat across the surfaces of the human body from a cohort of individuals. This would enable a more complete version of the type of comparison performed here.

10.1128/mSystems.00187-18.8 TABLE S5 Identification of taxa with a preference for the human nasal habitat using the SKn and SKs data sets. Download Table S5, XLSX file, 0.2 MB. Copyright © 2018 Escapa et al. 2018 Escapa et al. This content is distributed under the terms of the Creative Commons Attribution 4.0 International license.

Members of the genus *Corynebacterium* (phylum *Actinobacteria*) are common in human nasal, skin, and oral microbiomes, but their species-level distribution across these body sites remains less clear (23). Our analysis of the SKns clones identified three *Corynebacterium* species as primarily located in the nostrils compared to the other skin sites: *C. propinquum,* C. pseudodiphtheriticum, and *C. accolens* (Table S5). In the species-level reanalysis of the HMP nares V1-V3 data set, these species were among the top five *Corynebacterium* species/supraspecies by rank order abundance of sequences (Table S4B). In this reanalysis, Corynebacterium tuberculostearicum accounted for the fourth largest number of sequences; however, in the SKns clones, it was not disproportionately present in the nostrils. Therefore, although common in the nostrils, we did not consider the nostrils the primary habitat for *C. tuberculostearicum*, in contrast to *C. propinquum,* C. pseudodiphtheriticum, and *C. accolens.*

**(iv) The human skin and nostrils are the primary habitats of Lawsonella clevelandensis.** In 2016, Lawsonella clevelandensis was described as a novel genus and species within the suborder *Corynebacterineae* (phylum *Actinobacteria*) (49); genomes for two isolates are available (50). It was initially isolated from several human abscesses, mostly from immunocompromised hosts, but its natural habitat was unknown. This led to speculation that L. clevelandensis might be a member of the human microbiome or an environmental microbe with the capacity for opportunistic infection (49, 51). Our results indicate that *L. clevelandensis* is a common member of the bacterial microbiome of some oily skin sites and the nostrils of humans (Table S5). Indeed, in the SKn clones, we detected *L. clevelandensis* as the 11th most abundant taxon. Validating the SKn data in our reanalysis of the HMP nares V1-V3 data set from 210 participants, we found that *L. clevelandensis* was the 5th most abundant species overall with a prevalence of 86% (Table S4B). In the nostrils of individual HMP participants, *L. clevelandensis* had an average relative abundance of 5.7% and a median relative abundance of 2.6% (range, 0 to 42.9%). *L. clevelandensis* is recently reported to be present on skin (52). Our reanalysis of the SKns clones indicated that of these body sites, the primary habitat for *L. clevelandensis* is oily skin sites, in particular the alar crease, glabella, and occiput where it accounts for higher relative abundance than in the nostrils (Table S5). Virtually nothing is known about the role of *L. clevelandensis* in the human microbiome. It has been reported to grow best under anaerobic conditions (<1% O_2_), and cells are a mixture of pleomorphic cocci and bacilli that stain Gram variable to Gram positive and partially acid fast (49, 50). On the basis of its 16S rRNA gene sequence, *L. clevelandensis* is most closely related to the genus *Dietzia*, which includes mostly environmental species. Within its suborder *Corynebacterineae* are other genera associated with humans, including *Corynebacterium*, which is commonly found on oral, nasal, and skin surfaces, and *Mycobacterium.* Our analyses demonstrate that *L. clevelandensis* is a common member of the human skin and nasal microbiomes, opening up opportunities for future research on its ecology and its functions with respect to humans.

**(v) The majority of the bacteria detected in our reanalysis of the human nasal passages are cultivated.** Using blastn to compare the 16S rRNA gene SKn clones with eHOMDv15.1, we found that 93.1% of these sequences from adult nostrils can be assigned to cultivated named species, 2.1% to cultivated unnamed taxa, and 4.7% to uncultivated unnamed taxa. In terms of the total number of species-level taxa represented by the SKn clones, rather than the total number of sequences, 70.1% matched cultivated named taxa, 14.4% with cultivated unnamed taxa, and 15.5% with uncultivated unnamed taxa. Similarly, in the HMP nares V1-V3 data set from 210 participants (see below), 91.1% of sequences represented cultivated named bacterial species. Thus, the bacterial microbiota of the nasal passages is numerically dominated by cultivated bacteria. In contrast, approximately 30% of the oral microbiota (http://www.ehomd.org) and a larger, but not precisely defined, fraction of the intestinal microbiota are currently uncultivated (53, 54). The ability to cultivate the majority of species detected in the nasal microbiota is an advantage when studying the functions of members of the nasal microbiome.

**(vi) One common nasal taxon remains to be cultivated.** In exploring the SKn data set to generate eHOMD, we realized that the 12th most abundant clone in the SKn data set lacked genus-level assignment. To ensure this was not just a common chimera, we broke the sequence up into thirds and fifths and subjected each fragment to blastn against eHOMD and GenBank. The fragments hit only our reference sequences and were distant to other sequences across the entire length. Therefore, this clone represents an unnamed and apparently uncultivated *Neisseriaceae* bacterial taxon to which we have assigned the provisional name *Neisseriaceae* [G-1] bacterium HMT-174 (named G-1 for unnamed genus 1). Its provisional naming facilitates recognition of this bacterium in other data sets and its future study. In our reanalysis of the HMP nares V1-V3 data set, *Neisseriaceae* [G-1] bacterium HMT-174 was the 10th most abundant species overall with a prevalence of 35%. In individual participants, it had an average relative abundance of 1.3% and a median relative abundance of 0 (range, 0 to 38.4%). Blastn analysis of our reference sequence for *Neisseriaceae* [G-1] bacterium HMT-174 against the 16S rRNA sequence database at NCBI gave matches of 90% to 92% similarity to members of the family *Neisseriaceae* and matches to the neighboring family *Chromobacteriaceae* at 88% to 89%. A phylogenetic tree of taxon HMT-174 with members of these two families was more instructive, since it clearly placed taxon HMT-174 as a deeply branching, but monophyletic, member of the *Neisseriaceae* family with the closest named taxa being Snodgrassella alvi (NR_118404) at 92% similarity and Vitreoscilla stercoraria (NR_0258994) at 91% similarity, and the main cluster of *Neisseriaceae* at or below 92% similarity (Data S1E). The main cluster of genera in a tree of the family *Neisseriaceae* includes *Neisseria, Alysiella, Bergeriella, Conchiformibius, Eikenella, Kingella,* and other mammalian host-associated taxa. There is a separate clade of the insect-associated genera *Snodgrassella* and *Stenoxybacter*, whereas *Vitreoscilla* is from cow dung and forms its own clade. Recognition of the as-yet-uncultivated *Neisseriaceae* [G-1] bacterium HMT-174 as a common member of the adult nostril microbiome supports future research to cultivate and characterize this bacterium. *Neisseriaceae* [G-1] bacterium HMT-327 is another uncultivated nasal taxon, likely from the same unnamed genus, and the 20th (HMP) and 46th (SKn) most common nasal organism in the two data sets we reanalyzed. There are several additional uncultured nasal bacteria in eHOMD, highlighting the need for sophisticated cultivation studies even in the era of NGS studies. Having 16S rRNA reference sequences tied to the provisional taxonomic scheme in eHOMD allows targeted efforts to culture the previously uncultivated bacteria based on precise 16S rRNA identification methods.

**FIG 3:**
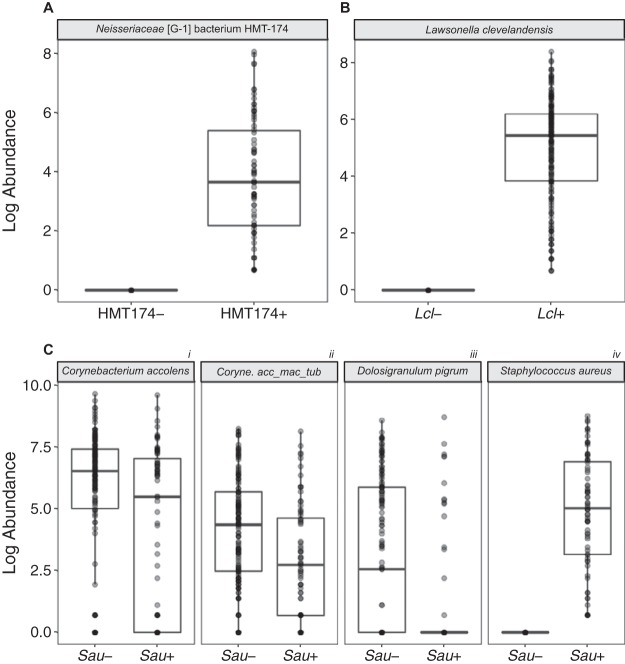
Three common nasal species/supraspecies exhibit increased differential relative abundance when S. aureus is absent from the nostril microbiome. In contrast, no other species showed differential abundance based on the presence or absence of *Neisseriaceae* [G-1] bacterium HMT-174 or Lawsonella clevelandensis. (A to C) We used ANCOM to analyze species/supraspecies-level composition of the HMP nares V1-V3 data set when *Neisseriaceae* [G-1] bacterium HMT-174 (A), (B) *L. clevelandensis* (*Lcl*) (B), or S. aureus (*Sau*) (C) was either absent (−) or present (+). Results were corrected for multiple testing. The dark bar represents the median, and lower and upper hinges correspond to the first and third quartiles. Each gray dot represents the value for a sample, and multiple overlapping dots appear black. *Coryne. acc_mac_tub* represents the supraspecies *Corynebacterium accolens_macginleyi_tuberculostearicum*.

**(vii) No species are differentially abundant with respect to either *Neisseriaceae* [G-1] bacterium HMT-174 or *L. clevelandensis*.** There is a lack of knowledge about potential relationships between the two newly recognized members of the nostril microbiome, *L. clevelandensis* and *Neisseriaceae* [G-1] bacterium HMT-174, and other known members of the nostril microbiome. Therefore, we performed analysis of composition of microbiomes, also known as ANCOM (55), on samples grouped based on the presence or absence of sequences of each of these two taxa of interest in search of species displaying differential relative abundance based on either one. For *Neisseriaceae* [G-1] bacterium HMT-174, this was targeted at identifying potential growth partners for this as-yet-uncultivated bacterium. However, ANCOM detected only the group-specific taxon in each case and did not reveal any other species with differential relative abundance with respect to either *Neisseriaceae* [G-1] bacterium HMT-174 (Fig. 3A) or *L. clevelandensis* (Fig. 3B).

**(viii) Several common species of nasal bacteria are more abundant when S. aureus is absent.** Finally, as proof of principle that eHOMD enhances the clinical relevance of 16S rRNA gene-based microbiome studies, we turned our attention to S. aureus, which is both a common member of the nasal microbiome and an important human pathogen, with >10,000 attributable deaths/year in the United States (56–58). The genus *Staphylococcus* includes many human commensals hence the clinical importance of distinguishing *aureus* from non-*aureus* species. In our reanalysis of the HMP nares V1-V3 data set, S. aureus sequences accounted for 3.9% of the total sequences with a prevalence of 34% (72 of the 210 participants), consistent with it being common in the nasal microbiome (2, 59). S. aureus nostril colonization is a risk factor for invasive infection at distant body sites (56, 60). Therefore, in the absence of an effective vaccine (61, 62), there is increasing interest in identifying members of the nostril and skin microbiome that might play a role in colonization resistance to S. aureus (e.g., references [Bibr B63][Bibr B64][Bibr B66]). Although differential relative abundance does not indicate causation, identifying such relationships at the species level in a cohort the size of the HMP can arbitrate variations among findings in smaller cohorts and generate new hypotheses for future testing. Therefore, we used ANCOM to identify taxa displaying differential relative abundance in HMP nostril samples in which 16S rRNA gene sequences corresponding to S. aureus were absent or present (55). In this HMP cohort of 210 adults, two *Corynebacterium* species/supraspecies––*accolens* and *accolens_macginleyi_tuberculostearicum*––showed positive differential abundance in the absence of S. aureus nostril colonization (Fig. 3C, panels i and ii). These two were among the nine most abundant species in the cohort overall (Fig. 2C and Table S4B). As previously reviewed (47), there is variability between studies with smaller cohorts with respect to the reported correlations between S. aureus and specific *Corynebacterium* species in the nostril microbiome; this variability might relate to strain-level differences and/or to the small cohort sizes. *D. pigrum* (67) also showed a positive differential abundance in the absence of S. aureus (Fig. 3C, panel iii). This is consistent with observations from Liu, Andersen, and colleagues that high levels of *D. pigrum* are the strongest predictor of the absence of S. aureus nostril colonization in 89 older adult Danish pairs of twins (68). In our reanalysis of the HMP nares V1-V3 data set, *D. pigrum* was the 6th most abundant species overall with a prevalence of 41% (Fig. 2C and Table S4B). There were no species other than the group-specific taxon S. aureus with positive differential abundance when S. aureus was present (Fig. 3C, panel iv).

**Summary.** As demonstrated here, the eHOMD (http://www.ehomd.org) is a comprehensive well-curated online database for the bacterial microbiome of the entire aerodigestive tract enabling species/supraspecies-level taxonomic assignment to full-length and V1-V3 16S rRNA gene sequences and including correctly assigned, annotated available genomes. In generating the eHOMD, we identified two previously unrecognized common members of the adult human nostril microbiome, opening up new avenues for future research. As illustrated using the adult nostril microbiome, eHOMD can be leveraged for species-level analyses of the relationship between members of the aerodigestive tract microbiome, enhancing the clinical relevance of studies, and generating new hypotheses about interspecies interactions and the functions of microbes within the human microbiome. The eHOMD provides a broad range of microbial researchers, from basic to clinical, a resource for exploring the microbial communities that inhabit the human respiratory and upper digestive tracts in health and disease.

## MATERIALS AND METHODS

### Generating the provisional eHOMDv15.01 by adding bacterial species from culture-dependent studies.

To identify candidate human microbial taxa (cHMTs), we reviewed two studies that included cultivation of swabs taken from along the nasal passages in both healthy individuals and individuals with chronic rhinosinusitis (CRS) (18, 19) and one study of mucosal swabs and nasal washes from healthy individuals only (17). We also reviewed a culture-dependent study of anaerobic bacteria isolated from cystic fibrosis (CF) sputum specimens to identify anaerobes that might be present in the nasal passages/sinuses in individuals with CF (20). Using this approach, we identified 162 cHMTs, of which 65 were present in HOMDv14.51 and 97 were not (Fig. 1A; see also Table S1A in the supplemental material). For each of these 97 named species, we downloaded at least one 16S rRNA gene RefSeq from NCBI 16S (via a search of BioProject accession numbers 33175 and 33317) (28) and assembled these into a reference database for blast. We then queried this via blastn with the SKn data set to determine which of the 97 cHMTs were either residents or very common transients of the nasal passages (Fig. 1A). We identified 30 cHMTs that were represented by ≥10 sequences in the SKn data set with a match at ≥98.5% identity. We added these 30 candidate taxa, represented by 31 16S rRNA gene reference sequences for eHOMD (eHOMDrefs), as permanent HMTs to the HOMDv14.51 alignment to generate eHOMDv15.01 (Fig. 1A and Table S6A). Of note, with the addition of nonoral taxa, we have replaced the old provisional taxonomy prefix of human oral taxon (HOT) with human microbial taxon (HMT), which is applied to all taxa in the eHOMD.

10.1128/mSystems.00187-18.9 TABLE S6 Summary of additions in the current expansion of HOMD in order to generate eHOMDv15.1, including new eHOMDrefs added to both new and existing HMTs (A) and newly added genomes (B). Download Table S6, XLSX file, 0.03 MB. Copyright © 2018 Escapa et al. 2018 Escapa et al. This content is distributed under the terms of the Creative Commons Attribution 4.0 International license.

### Generating the provisional eHOMDv15.02 by identifying additional HMTs from a data set of 16S rRNA gene clones from human nostrils.

For the second step in expanding the HOMD, we focused on obtaining new eHOMDrefs from the SKn data set (i.e., the 44,374 16S rRNA gene clones from nostril [anterior nares] samples generated by Julie Segre, Heidi Kong, and colleagues [11–16]). We used blastn to query the SKn clones versus the provisional database eHOMDv15.01. Of the nostril-derived 16S rRNA gene clones, 37,716 of 44,374 matched reference sequences in eHOMDv15.01 at ≥98.5% identity (Fig. 1B), and 6,163 matched reference sequences in eHOMDv15.01 at <98% (Fig. 1C). The SKn clones that matched eHOMDv15.01 at ≥98.5% could be considered already identified by eHOMDv15.01. Nevertheless, these clones were used as queries to perform blastn searches versus the NCBI 16S database (28) to identify other NCBI RefSeqs that might match these clones with better identity. We compared the blastn results against eHOMDv15.01 and NCBI 16S and if the match was substantially better to a high-quality sequence (close to full-length and without unresolved nucleotides) from the NCBI 16S database, then that one was considered for addition to the database. Using this approach, we identified two new HMTs (represented by one eHOMDref each) and five new eHOMDrefs for taxa present in eHOMDv14.51 that improved capture of sequences to these taxa (Fig. 1B and Table S6A). For the 6,163 SKn clones that matched reference sequences in eHOMDv15.01 at <98%, we performed clustering at ≥98.5% identity across 99% coverage and inferred an approximately maximum likelihood phylogenetic tree (Fig. 1C and see Text S1 in the supplemental material). If a cluster (an M-OTU) had ≥10 clone sequences (30 out of 32), then we chose a representative sequence(s) from that cluster based on a visual assessment of the cluster alignment. Each representative sequence was then queried against the NCBI nr/nt database to identify either the best high-quality, named species-level match or, lacking this, the longest high-quality clone sequence to use as the eHOMDref. Clones lacking a named match were assigned a genus name based on their position in the tree and an HMT number, which serves as a provisional name. The cluster representative sequence(s) plus any potentially superior reference sequences from the NCBI nr/nt database were finally added to the eHOMDv15.01 alignment to create the eHOMDv15.02. Using this approach, we identified and added 28 new HMTs, represented in total by 38 eHOMDrefs (Fig. 1C and Table S6A). Of note, we set aside the 1.1% (495 of 44,374) of SKn clones that matched at between 98 and 98.5% identify to avoid calling a taxon where no new taxon existed in the tree-based analysis of sequences that matched at <98%.

### Generating the provisional eHOMDv15.03 by identifying additional candidate taxa from culture-independent studies of aerodigestive tract microbiomes.

To further improve the performance of the evolving eHOMD, we took all of the SKn data set clones that matched eHOMDv15.02 at <98.5% identity, clustered these at ≥98.5% identity across a coverage of 99%, and inferred an approximately maximum likelihood phylogenetic tree (Text S1). Subsequent evaluation of this tree (see previous section) identified two more HMTs (represented in total by three eHOMDrefs) and one new eHOMDref for a taxon already in the database for addition to eHOMDv15.03 (Fig. 1D and Table S6A). To identify additional taxa that are resident in sites in the aerodigestive tract beyond the mouth and that are not represented by enough clones in the SKn data set to meet our criteria, we iteratively evaluated the performance of eHOMDv15.02 with five other 16S rRNA gene data sets from aerodigestive tract sites outside the mouth (Fig. 1E). We used the following criteria to select these data sets to assay for the performance of eHOMDv15.02 as a reference database for the aerodigestive tract across the life span of healthy humans and humans with disease. (i) All sequences covered at least variable regions 1 and 2 (V1-V2), because for many bacteria resident in the aerodigestive tract, V1-V2/V1-V3 includes sufficient sequence variability to obtain species-level assignment (Table 3). (ii) The raw sequence data were either publicly available or readily supplied by the authors upon request. This approach yielded a representative set of data sets (Table S1C) (21–23, 25–27). Additional information on how we obtained and prepared each data set for use is in Text S1. For each data set from Table S1C, we separately performed a blastn search against eHOMDv15.02 and filtered the results to identify the percentage of reads matching at ≥98.5% identity (Fig. 1E). To compare the performance of eHOMDv15.02 with other commonly used 16S rRNA gene databases, we also performed a blastn search against NCBI 16S (28), RDP16 (29), and SILVA128 (30, 31) databases using the same filter as with eHOMDv15.02 for each data set (Table S1C). If one of these other databases captured more sequences than eHOMDv15.02 at ≥98.5% identity, we then identified the reference sequence in the outperforming database that was capturing those sequences and evaluated it for inclusion in eHOMD. On the basis of this comparative approach, we added three new HMTs (represented by one eHOMDref each) plus five new eHOMDrefs for taxa already present in eHOMDv15.02 to the provisional database to create eHOMDv15.03 (Fig. 1E and Table S6A).

### Generating the provisional eHOMDv15.04 by identifying additional candidate taxa from a data set of 16S rRNA gene clones from human skin.

Having established that eHOMDv15.03 serves as an excellent 16S rRNA gene database for the aerodigestive tract microbiome in healthy and diseased humans, we were curious as to how it would perform when evaluating 16S rRNA gene clone libraries from skin sites other than the nostrils. As reviewed in reference 47, in humans, the area just inside the nostrils, which are the openings into the nasal passages, is the skin-covered surface of the nasal vestibule. Prior studies have demonstrated that the bacterial microbiota of the skin of the nasal vestibule (also known as nostrils or nares) is distinctive and most similar to other moist skin sites (11). To test how well eHOMDv15.03 performed as a database for skin microbiota in general, we executed a blastn search using 16S rRNA gene clones from all of the nonnasal skin sites included in the Segre-Kong data set (SKs) to assess the percentage of total sequences captured at ≥98.5% identity over ≥98% coverage. Only 81.7% of the SKs clones were identified with eHOMDv15.03, whereas 95% of the SKn clones were identified (Table S1B). We took the unidentified SKs sequences and did blastn searches versus the SILVA128 database with the same filtering criteria. To generate eHOMDv15.04, we first added the top 10 species from the SKs data set that did not match to sequences in eHOMDv15.03, all of which had >350 reads in SKs (Fig. 1F and Table S6A). Of note, for two of the skin-covered body sites, a single taxon accounted for the majority of reads that were unassigned with eHOMDv15.03: Staphylococcus auricularis from the external auditory canal and Corynebacterium massiliense from the umbilicus. Addition of these two taxa considerably improved the performance of eHOMD for their respective body site. Next, we revisited the original list of 97 cHMTs and identified 4 species that are present in ≥3 of the 34 subjects in the study of Kaspar et al. (19) (Table S1A, column E) that had ≥30 reads in the SKs data set and that matched sequences in SILVA128 but not to sequences in eHOMDv15.03. We added these species to generate eHOMDv15.04 (Fig. 1G and Table S6A).

### Establishing eHOMD reference sequences and final updates to generate eHOMDv15.1.

Each eHOMD reference sequence (eHOMDref) is a manually corrected representative sequence with a unique alphanumeric identifier that starts with its three-digit HMT number; each is associated with the original NCBI accession number of the candidate sequence. For each candidate 16S rRNA gene reference sequence selected, a blastn search was performed against the NCBI nr/nt database and filtered for matches at ≥98.5% identity to identify additional sequences for comparison in an alignment, which was used to either manually correct the original candidate sequence or select a superior candidate from within the alignment. Manual correction included correction of all ambiguous nucleotides, any likely sequencing miscalls/errors, and addition of consensus sequence at the 5′/3′ ends to achieve uniform length. All ambiguous nucleotides from earlier versions were corrected in the transition from HOMDv15.04 to eHOMDv15.1 because ambiguous bases, such as “R” and “Y,” are always counted as mismatches against a nonambiguous base. Also, in preparing eHOMDv15.1, nomenclature for *Streptococcus* species was updated in accordance with reference 69, and genus names were updated for species that were formerly part of the *Propionibacterium* genus in accordance with reference 70. *Cutibacterium* is the new genus name for the formerly cutaneous *Propionibacterium* species (70). In addition to the 79 taxa added in the expansion from HOMDv14.51 to eHOMDv15.04 (Table S6A), 4 oral taxa were added to the final eHOMDv15.1: *Fusobacterium hwasookii* HMT-953, *Saccharibacteria* (TM7) bacterium HMT-954, *Saccharibacteria* (TM7) bacterium HMT-955, and Neisseria cinerea HMT-956. Also, *Neisseria pharyngis* HMT-729 was deleted because it is not validly named and is part of the *Neisseria sica-N. mucosa-N. flava* complex.

### Identification of taxa with a preference for the human nasal habitat.

We assigned 13 taxa as having the nostrils as their preferred body site habitat. To achieve this, we first performed the following steps as illustrated in Table S5. (i) We performed blastn searches of SKn and SKs versus eHOMDv15.04 and used the first hit based on E value to assign putative taxonomy to each clone. (ii) We used these names to generate a count table of taxa and body sites. (iii) We normalized the total number of clones per body site to 20,000 each for comparisons (columns B to V). (iv) For each taxon, we used the total number of clones across all body sites as the denominator (column W) to calculate the percentage of that clone present at each specific body site (columns Z to AT). (v) We calculated the ratio of the percentage of each taxon in the nostrils to the expected percentage if that taxon was evenly distributed across all 21 body sites in the SKns clone data set (column Y). (vi) We sorted all taxa in Table S5 by rank abundance among the nostril clones (column X). Finally, of the top 20 taxa, we assigned nasal as the preferred body site to those taxa that were elevated ≥2× in the nostrils versus what would be expected if evenly distributed across all the skin sites (column Y). This conservative approach established a lower bound for the eHOMD taxa that have the nasal passages as their preferred habitat. The SKn data set includes samples from children and adults who are healthy and those who have a disease (11–16). In contrast, the HMP nares V1-V3 data are from healthy adults aged 18 to 40 years (23, 24). Of the species classified as nasal in eHOMDv15.01, 8 of the 13 are in the top 19 most abundant species from the 210-person HMP nares V1-V3 data set.

### Reanalysis of the HMP nares V1-V3 data set to the species level.

We aligned the 2,338,563 chimera-cleaned reads present in the HMPnV1-V3 (Text S1) in QIIME 1 (align_seqs.py with the default method; PyNAST) (71, 72), using eHOMDv15.04 as the reference database and trimmed for MED using “o-trim-uninformative-columns-from-alignment” and “o-smart-trim” scripts (3). A total of 2,203,471 reads (94.2% of starting) were recovered after the alignment and trimming steps. After these initial cleaning steps, samples were selected such that only those with more than 1,000 reads were retained and each subject was represented by only one sample. For subjects with more than one sample in the total HMP nares V1-V3 data, we selected for use the one with more reads after the cleaning steps to avoid bias. Thus, what we refer to as the HMP nares V1-V3 data set included 1,627,514 high-quality sequences representing 210 subjects. We analyzed this data set using MED with minimum substantive abundance of an oligotype (-M) equal to 4 and maximum variation allowed in each node (-V) equal to 12 nt, which equals 2.5% of the 820-nucleotide length of the trimmed alignment. Of the 1,627,514 sequences, 89.9% (1,462,437) passed the -M and -V filtering and are represented in the MED output. Oligotypes were assigned taxonomy in R with the dada2::assignTaxonomy() function (an implementation of the RDP naive Bayesian classifier algorithm with a kmer size of 8 and a bootstrap of 100) (4, 42) using the eHOMDv15.1 V1-V3 Training Set (version 1). We then collapsed oligotypes within the same species/supraspecies yielding the data shown in Table S7. The count data in Table S7 were converted to relative abundance by samples at the species/supraspecies level to generate an input table for ANCOM, including all identified taxa (i.e., we did not remove taxa with low relative abundance). ANCOM (version 1.1.3) was performed using the presence or absence of *Neisseriaceae* [G-1] bacterium HMT-174, *L. clevelandensis,* or S. aureus as group definers. ANCOM default parameters were used (sig = 0.05, tau = 0.02, theta = 0.1, repeated = FALSE) except that we performed a correction for multiple comparisons (multcorr = 2) instead of using the default no correction (multcorr = 3) (55).

10.1128/mSystems.00187-18.10 TABLE S7 Table of counts per sample and taxa in the HMP nares V1-V3 data set result of the reanalysis at the species/supraspecies level. Download Table S7, XLSX file, 0.3 MB. Copyright © 2018 Escapa et al. 2018 Escapa et al. This content is distributed under the terms of the Creative Commons Attribution 4.0 International license.

### Recruitment of genomes matching HMTs to eHOMD and assignment of species-level names to genomes previously named only to the genus level.

Genomic sequences were downloaded from the NCBI FTP site (ftp://ftp.ncbi.nlm.nih.gov/genomes). Genome information, e.g., genus, species, and strain name, were obtained from a summary file listed on the FTP site in July 2018: ftp://ftp.ncbi.nlm.nih.gov/genomes/ASSEMBLY_REPORTS/assembly_summary_genbank.txt. To recruit genomes for provisionally named eHOMD taxa (HMTs), genomic sequences from the same genus were targeted. For six genera present in eHOMD, we downloaded and analyzed 130 genomic sequences from GenBank that were taxonomically assigned only to the genus level (i.e., with “sp.” in the species annotation) because some of these might belong to a HMT. To determine the closest HMT for each of these genomes, the 16S rRNA genes were extracted from each genome and were blastn searched against the eHOMDv15.1 reference sequences. Of the 130 genomes tested, we excluded 13 that had <98% sequence identity to any of the eHOMDrefs. The remaining 117 genomes fell within a total of 25 eHOMD taxa at a percent identity of ≥98.5 to one of the eHOMDrefs (Table S6B). To validate the phylogenetic relatedness of these genomes to HMTs, the extracted 16S rRNA gene sequences were then aligned with the eHOMDrefs using MAFFT software (V7.407) (73), and a phylogenetic tree was generated using FastTree (version 2.1.10.Dbl) (74) with the default Jukes-Cantor + CAT model for tree inference (Data S1C). The relationship of these genomes to eHOMD taxa was further confirmed by performing phylogenomic analysis in which all the protein sequences of these genomes were collected and analyzed using PhyloPhlAn, which infers a phylogenomic tree based on the most-conserved 400 bacterial protein sequences (41) (Data S1D). These 117 genomes were then added to the eHOMDv15.1 as reference genomes. At least one genome from each taxon is dynamically annotated against a frequently updated NCBI nonredundant protein database so that potential functions may be assigned to hypothetical proteins due to matches to newly added proteins with functional annotations in the NCBI nr database.

### Data availability.

Links to the external data sets and databases used are available in Text S1 in the supplemental material. The most up-to-date version of eHOMD is available for download via a link at http://www.ehomd.org or at http://ehomd.org/index.php?name=Download. Any code or other data needed to reproduce the results of this paper will be made available upon reasonable request.
