# Supplementary material for: New Insights into Human Nostril Microbiome from the Expanded Human Oral Microbiome Database (eHOMD): a Resource for the Microbiome of the Human Aerodigestive Tract
Source: mSystems. 2018 Dec 4;3(6):e00187-18. doi: 10.1128/mSystems.00187-18 (PMC6280432; doi:10.1128/mSystems.00187-18)
Supplement: DATA S1 [file sys006182299sd1.docx]

**SUPPLEMENTAL DATA S1**

New insights into human nostril microbiome from the *expanded* Human Oral Microbiome Database (*e*HOMD): a resource for species-level identification of microbiome data from the aerodigestive tract

Isabel F. Escapa^a,b^, Tsute Chen^a,b*^, Yanmei Huang^a,b*^, Prasad Gajare^a^, Floyd E. Dewhirst^a,b^, Katherine P. Lemon^a,c#^

**S1A. 16S rRNA gene phylogenetic tree of all of the *e*HOMD reference sequences (*e*HOMDrefs) in v15.1** (available online at <http://www.homd.org/ftp/publication_data/20180919/Supplemental_Figures/Figure.S1A>) The 998 16S rRNA gene references sequences were aligned with MAFFT (V7.047) and then subjected to FastTree (version 2.1.10.Dbl) to build a phylogenetic tree. The 111 newly added sequences (from 94 taxa) are highlighted in yellow. For each sequence the following information is provided and separated with a vertical bar “|”: 1) HMT ID (in blue), 2) sequence ID, 3) scientific name, 4) clone ID, 5) Genbank ID on which the sequence was based, 6) naming status (i.e., named or unnamed phylotype) and 7) body site, if assigned. The latest version of the *e*HOMD phylogenetic tree is available at <http://www.ehomd.org/ftp/HOMD_phylogeny/current>.

**S1B. 16S rRNA gene tree of *Corynebacterium* reference sequences from both SILVA128 and *e*HOMDv15.1** (available online at <http://www.homd.org/ftp/publication_data/20180919/Supplemental_Figures/Figure.S1B>) This tree shows all of the SILVA *Corynebacterium* sequences (sequence ID in red) clustered together with the *e*HOMDv15.1 *Corynebacterium* reference sequences (prefixed with HMT ID in blue and refseq ID in brown). SILVA sequences discussed in main text are highlighted in yellow and mostly near the bottom of the tree. To generate the tree, we aligned the 1,359 *Corynebacterium* reference sequences from SILVA128 together with the v15.1 *Corynebacterium* *e*HOMDRefs using MAFFT (v7.407) and used the aligned sequences to generate a tree with FastTree (version 2.1.10.Dbl). We included several *e*HOMD sequences from neighboring genera as an outgroup (top of tree). Some of the SILVA128 sequences have deep long branches, e.g., KP214641.3.1224 and CP001601.1487755.1489023. These are mostly due to chimeric sequences some of which include non-16S rRNA fragments (e.g., in the case of CP001601.1487755.1489023 only the first 906 of 1207 nucleotides match to 16S rRNA by blastn).

**S1C. Phylogenetic tree of 16S rRNA genes from newly added genomes** (available online at <http://www.homd.org/ftp/publication_data/20180919/Supplemental_Figures/Figure.S1C>) The annotated 16S rRNA gene sequences were extracted from the 117 newly added genomes and were aligned and treed together with the *e*HOMDv15.1 reference sequences to illustrate their phylogenetic positions amongst the sequences of known taxa. If a genome had multiple 16S rRNA gene sequences annotated, only the one with the highest sequence percent identity was included and highlighted in light green color. Taxon assignment was based on one or more of the following, with icons adjacent to each entry indicating which were used: 1) highest percent sequence identity to the *e*HOMDrefs v15.1 (blue diamond); 2) phylogenetic position of the 16S rRNA gene sequence from #1 (light green triangle); and 3) phylogenomic position in Fig. S5 (light orange circle). Other useful genomic information provided is explained in the figure key. The same genome IDs in the format of SEQFNNNN (where NNNN is a four-digit number) were denoted in both Fig. S4 and S5 for consistency.

**S1D. Phylogenomic tree of the newly added genomes** (available online at <http://www.homd.org/ftp/publication_data/20180919/Supplemental_Figures/Figure.S1D>) The annotated protein sequences were extracted from the 117 newly added genomes and subjected to phylogenomic analysis with PhyloPhlAn (version 0.99) to illustrate their phylogenetic positions amongst the sequences of known taxa. Newly added genomes are highlighted in light orange. Taxonomy assignment was based on one or more of the following, with icons adjacent to each added genome indicating which were used:1) highest percent sequence identity to the v15.1 *e*HOMDrefs (blue diamond); 2) phylogenetic position of the 16S rRNA gene sequence from #1 in Fig. S4 (light green triangle); and 3) phylogenomic positions in this figure (light orange circle). Other useful genomic information provided is explained in the figure key. The same genome IDs in the format of SEQFNNNN (where NNNN is a four-digit number) are denoted in both Fig. S4 and S5 for consistency.

**S1E. Phylogenetic tree of *Betaproteobacteria* showing the positions of *Neisseriaceae* [G-1] bacterium HMT-174 and HMT-327** (available online at <http://www.homd.org/ftp/publication_data/20180919/Supplemental_Figures/Figure.S1E>) To see where the novel genus *Neisseriaceae* [G-1] fell relative other taxa at the family, class and order level, 10 non-oral sequences (in black font) were added to *e*HOMD sequences (in blue and red font) from the class *Betaproteobacteria* and a phylogenetic tree was generated*.* Species were selected from the families *Neisseriaceae* and *Chromobacteriaceae* (the two families in the order *Neisseriales*) because some of these sequences were best hits by simple blastn analysis of the novel *Neisseriaceae* [G-1] species. The tree was generated by first aligning the sequences with the MAFFT software (V7.407) and then subjecting them to FastTree (Version 2.1.10.Dbl) with the default Jukes-Cantor + CAT model for inferring the tree. The scale bar represents substitutions/site. Order names are marked above the appropriate node and *Neisseriales* families are indicated with brackets.
