# Supplementary material for: New Insights into Human Nostril Microbiome from the Expanded Human Oral Microbiome Database (eHOMD): a Resource for the Microbiome of the Human Aerodigestive Tract
Source: mSystems. 2018 Dec 4;3(6):e00187-18. doi: 10.1128/mSystems.00187-18 (PMC6280432; doi:10.1128/mSystems.00187-18)
Supplement: TEXT S1 [file sys006182299s1.docx]

**SUPPLEMENTAL METHODS**

New insights into human nostril microbiome from the *expanded* Human Oral Microbiome Database (*e*HOMD): a resource for species-level identification of microbiome data from the aerodigestive tract

Isabel F. Escapa^a,b^, Tsute Chen^a,b*^, Yanmei Huang^a,b*^, Prasad Gajare^a^, Floyd E. Dewhirst^a,b^, Katherine P. Lemon^a,c#^

**Information on the aerodigestive tract microbiome datasets used.**

Segre, Kong and colleagues have deposited close-to-full-length 16S RNA gene sequences from clone libraries collected from different skin sites, including the nostrils (nares) at NCBI under BioProjects PRJNA46333 and PRJNA30125 ([1-6](#_ENREF_1)). We downloaded a total of 413,606 sequences from these BioProjects on May 11, 2017. The sequences were screened for bacterial 16S rRNA gene sequences only and parsed into two datasets: the SK nostril dataset (SKn), which includes 44,374 sequences from nostril samples with a mean length of 1354 bp (min. 1233, max. 1401); and the SK skin dataset (SKs), which includes 362,313 sequences with a mean length of 1356 bp (min. 1161, max. 1410). The SKs dataset includes 16S rRNA clone sequences derived from 20 non-nasal skin sites, including the alar crease, antecubital fossa, axillary vault, back, buttock, elbow, external auditory canal, glabella, gluteal crease, hypothenar palm, inguinal crease, interdigital web space, manubrium, occiput, plantar heel, popliteal fossa, retroauricular crease, toe web space, umbilicus and volar forearm.

The Human Microbiome Project (HMP) Data Coordination Center performed baseline processing and analysis of all 16S rRNA gene variable region sequences generated from >10,000 samples from healthy human subjects ([7](#_ENREF_7), [8](#_ENREF_8)). Table “HM16STR_healthy.csv” summarizes all the information for the 9811 files included in the dataset (<https://www.hmpdacc.org/hmp/HM16STR/healthy/>). We downloaded the 586 files labelled "anterior_nares" from the corresponding url identified in the same table. The downloaded files contain V1-V3, V3-V5 and V6-V9 data, therefore the reads were filtered based on the primer information recorded in each read header, resulting in a total of 3,458,862 "anterior_nares" V1-V3 reads corresponding to 363 samples from 227 subjects. (See Methods for why the cohort used for species-level reanalysis included 210 subjects). We selected the 2,351,347 reads (67.9%) with length ≥430 and ≤652 bp (the range of the V1-V3 16S rRNA gene region in HOMDv14.51). After *de novo* chimera removal with UCHIME in QIIME 1 ([9](#_ENREF_9), [10](#_ENREF_10)) (identify_chimeric_seqs.py -m usearch61) , there were 2,338,563 sequences for use. This dataset, dubbed HMPnV1-V3, was the starting point used to query the performance of the provisional versions of *e*HOMD and was the input for species-level reanalysis (see Methods).

Laufer et al. analyzed nostril swabs collected from 108 children ages 6 to 78 months in Philadelphia, PA between December 9, 2008 and January 2, 2009 for cultivation of *Streptococcus pneumoniae* and DNA harvest ([11](#_ENREF_11)). Of these, 44% were culture positive for *S. pneumoniae* and 23% were diagnosed with otitis media. 16S rRNA gene V1-V2 sequences were generated using Roche/454 with primers 27F and 338R. We obtained 184,685 sequences from the authors, of which 94% included sequence matching primer 338R and 1% included sequence matching primer 27F. Therefore, we performed demultiplexing in QIIME 1 (split_libraries.py) filtering reads for those ≥250 bp in length, quality score ≥30 and with barcode type hamming_8. **We also eliminated sequences from samples for which there was no metadata (n=108 for metadata) leaving** 120,963 sequences on which we performed *de novo* chimera removal with UCHIME in QIIME 1 (identify_chimeric_seqs.py -m usearch61) ([9](#_ENREF_9), [10](#_ENREF_10)), yielding the 120,274 16S rRNA V1-V2 sequences used here.

Allen et al. collected nasal lavage fluid samples from 10 participants before, during and after experimental nasal inoculation with rhinovirus ([12](#_ENREF_12)). 16S rRNA V1-V3 sequences were generated using 454-FLX platform and primers 27F and 534R. We obtained 99,095 sequences from the authors of which 77,322 (78%) passed a length filter of ≥300 bp. After *de novo* chimera removal in with UCHIME in QIIME 1 (identify_chimeric_seqs.py -m usearch61) ([9](#_ENREF_9), [10](#_ENREF_10)), there were 75,310 sequences for use in this study.

Pei et al. (2004) collected distal esophageal biopsies from four participants undergoing esophagogastroduodenoscopy for upper gastrointestinal complaints whose samples showed healthy esophageal tissue without evidence of pathology ([13](#_ENREF_13)). From each of these, they generated ten 16s rRNA gene clone libraries from independent amplifications using two different primer pairs: 1) 318 to 1,519 with inosine at ambiguous positions and 2) from 8 to 1513. Pei et al. (2005) also collected esophageal biopsies from 24 patients (9 with normal esophageal mucosa, 12 with gastroesophageal reflux disease (GERD), and 3 with Barrett’s esophagus) ([14](#_ENREF_14)). The Pei et al. 2004-2005 dataset also include all the novel sequences deposited in GenBank from this subsequent study. We downloaded a total of 7,414 close-to-full-length 16S rRNA gene sequences from GenBank (GB: DQ537536.1 to DQ537935.1 and DQ632752.1 to DQ639751.1 (PopSet 109141097), AY212255.1 to AY212264.1 (PopSet 28894245), AY394004.1, AY423746.1, AY423747.1 and AY423748.1).

Harris et al. collected bronchoalveolar lavage fluid from children with cystic fibrosis and generated 16S rRNA clone libraries from these ([15](#_ENREF_15)). We downloaded these 3203 clones from GenBank (GB: EU111806.1 to EU112454.1 (PopSet 157058892), DQ188268.1 to DQ188805.1 (PopSet 77819181) and AY805987.1 to AY808002.1 (PopSet 60499797)).

van der Gast et al. generated 16S rRNA gene clone libraries from spontaneously expectorated sputum samples collected from 14 adults with cystic fibrosis ([16](#_ENREF_16)). We downloaded these 2137 clones from GenBank (GB: FM995625.1 to FM997761.1).

Flanagan et al. generated 16S rRNA gene clone libraries from daily endotracheal aspirates collected from seven intubated patients ([17](#_ENREF_17)). We downloaded these 3278 clones from GenBank (GB: EF508731.1 to EF512008.1).

Perkins et al. collected endotracheal tubes from eight adults with mechanical ventilation to generate 16S rRNA gene clone libraries ([18](#_ENREF_18)). We downloaded these 1263 clones from GenBank (GB: FJ557249.1 to FJ558511.1).

**Information on the 16S rRNA gene databases used.**

The NCBI 16S Microbial database (NCBI 16S) was downloaded from <ftp://ftp.ncbi.nlm.nih.gov/blast/db/> on May 28, 2017 ([19](#_ENREF_19)). RDP16 (rdp_species_assignment_16.fa.gz) and SILVA128 (silva_species_assignment_v128.fa.gz) files were downloaded from <https://benjjneb.github.io/dada2/training.html> and converted to BLAST databases using “makeblastdb” from the NCBI blast 2.6.0+ package (<https://www.ncbi.nlm.nih.gov/books/NBK279690/>) ([20-22](#_ENREF_20)).

Greengenes GOLD was used instead of Greengenes because only 22.6% of 16S rRNA gene sequences in Greengenes had complete taxonomic information to the species level, whereas for 77.4% of the sequences the 7^th^ (species) level was listed simply as “s__”. In contrast, in Greengenes GOLD all sequences included 7 levels of taxonomic information, as needed for species-level identification. The Greengenes GOLD was downloaded from <http://greengenes.lbl.gov/Download/Sequence_Data/Fasta_data_files/gold_strains_gg16S_aligned.fasta.gz>. The total number of sequences in the database is 5441 (six of the entries in the fasta file consisted only of a header without data, thus were removed). The aligned fasta file was converted to a nonaligned file by removing all "." and "-", and further converted to a BLAST database using “makeblastdb” as above.

**Addition of 16S rRNA sequences to the *e*HOMD alignment.**

*e*HOMD maintains an alignment of all its reference 16S rRNA sequences. This alignment is based on the 16S rRNA secondary structure and is performed manually on a custom sequence editor (written in QuickBasic and available from Floyd E. Dewhirst at fdewhirst@forsyth.org). The corresponding alignment, in phylogenetic order, for each release of HOMD/*e*HOMD can be downloaded at <http://www.homd.org/?name=seqDownload&type=R>.

**Clustering sequences at ≥98.5% and generating phylogenetic trees.**

We performed blastn with an all-by-all search of the input sequences (Fig. 1C and 1D). The blastn results were used to cluster the sequences into operational taxonomic units (OTUs) based on percent sequence identity and alignment coverage. Specifically, all sequences were first sorted by size (seq_sort_len.fasta) in descending order and binned into operational taxonomic units (OTUs) at ≥98.5% identity across ≥99% coverage from longest to shortest sequences. If any subsequent sequence matched a previous sequence at ≥98.5% with coverage of ≥99%, the subsequent sequence was binned together with the previous sequence. If the subsequent sequence did not match any previous sequence, it was placed in new bin (i.e., 98.5% OTU). If the subsequent sequences matched multiple previous sequences that belong to more than one OTU, the subsequent sequence was binned to multiple OTUs, and at the same time, we formed a meta-OTU (M-OTU) linking these OTUs together. Next, we extracted sequences from each M-OTU and saved to individual fasta files. We then performed sequence alignment using software MAFFT ([23](#_ENREF_23)) (V7.407) for each M-OTU fasta file and constructed phylogenetic trees for each M-OTU. The trees were built using FastTree (v2.1.10.Dbl), which estimates nucleotide evolution with the Jukes-Cantor model and infers phylogenetic trees based on approximately maximum-likelihood ([24](#_ENREF_24)). We organized the trees by using the longest branch as root and ordered from fewest nodes to more subnodes.

**Additional information for candidate HMTs (cHMTs).**

Of the 97 cHMTs for addition to HOMD, 82 are present in a nasal culturome of 34 participants (Table S1A, column E), 18 with evidence of chronic nasal inflammation and 16 without evidence of nasal/systemic inflammation, based on swabs taken during nasal surgery from the anterior and posterior nasal vestibule (skin surface inside the nostrils) and the inferior and middle meatuses ([25](#_ENREF_25)). Of the other 15 cHMTs we found 7 only in a report of cultivation of intraoperative mucosal swabs from 38 participants with chronic rhinosinusitis (CRS) versus 6 controls ([26](#_ENREF_26)); 7 only in sputa from 50 adults with CF ([27](#_ENREF_27)); and 1 only in a report of the aerobic bacteria collected via a mucosal swab of the inferior turbinate and via a nasal wash from each of 10 healthy adults ([28](#_ENREF_28)).

**Evaluation of Computational Efficiency**

We randomly extracted ten 16S rRNA gene full length reads from the SKn dataset for use as query in a blastn vs. the different databases. We ran the blast 2.6.0+ command: “blastn -db YOURDATABASEHERE -query YOURQUERYFILEHERE -out OUTPUT.txt -outfmt "10 std qcovs salltitles" -max_target_seqs 1” using a single processor thread on a computer with the Intel Xeon CPU (X5675 @ 3.07GHZ with 24 Gb memory).  We used Linux shell command “time” before the blastn command to record the running time.

**References for the Supplemental Methods**

1. Grice EA, Kong HH, Conlan S, Deming CB, Davis J, Young AC, Bouffard GG, Blakesley RW, Murray PR, Green ED, Turner ML, Segre JA. 2009. Topographical and temporal diversity of the human skin microbiome. Science 324:1190-2.

2. Kong HH, Oh J, Deming C, Conlan S, Grice EA, Beatson MA, Nomicos E, Polley EC, Komarow HD, Murray PR, Turner ML, Segre JA. 2012. Temporal shifts in the skin microbiome associated with disease flares and treatment in children with atopic dermatitis. Genome research 22:850-9.

3. Oh J, Conlan S, Polley EC, Segre JA, Kong HH. 2012. Shifts in human skin and nares microbiota of healthy children and adults. Genome medicine 4:77.

4. Findley K, Oh J, Yang J, Conlan S, Deming C, Meyer JA, Schoenfeld D, Nomicos E, Park M, Kong HH, Segre JA. 2013. Topographic diversity of fungal and bacterial communities in human skin. Nature 498:367-70.

5. Oh J, Freeman AF, Park M, Sokolic R, Candotti F, Holland SM, Segre JA, Kong HH. 2013. The altered landscape of the human skin microbiome in patients with primary immunodeficiencies. Genome research 23:2103-14.

6. Oh J, Byrd AL, Deming C, Conlan S, Kong HH, Segre JA. 2014. Biogeography and individuality shape function in the human skin metagenome. Nature 514:59-64.

7. Human Microbiome Project C. 2012. Structure, function and diversity of the healthy human microbiome. Nature 486:207-14.

8. Human Microbiome Project C. 2012. A framework for human microbiome research. Nature 486:215-21.

9. Caporaso JG, Kuczynski J, Stombaugh J, Bittinger K, Bushman FD, Costello EK, Fierer N, Pena AG, Goodrich JK, Gordon JI, Huttley GA, Kelley ST, Knights D, Koenig JE, Ley RE, Lozupone CA, McDonald D, Muegge BD, Pirrung M, Reeder J, Sevinsky JR, Turnbaugh PJ, Walters WA, Widmann J, Yatsunenko T, Zaneveld J, Knight R. 2010. QIIME allows analysis of high-throughput community sequencing data. Nat Methods 7:335-6.

10. Edgar RC, Haas BJ, Clemente JC, Quince C, Knight R. 2011. UCHIME improves sensitivity and speed of chimera detection. Bioinformatics 27:2194-200.

11. Laufer AS, Metlay JP, Gent JF, Fennie KP, Kong Y, Pettigrew MM. 2011. Microbial communities of the upper respiratory tract and otitis media in children. MBio 2:e00245-10.

12. Allen EK, Koeppel AF, Hendley JO, Turner SD, Winther B, Sale MM. 2014. Characterization of the nasopharyngeal microbiota in health and during rhinovirus challenge. Microbiome 2:22.

13. Pei Z, Bini EJ, Yang L, Zhou M, Francois F, Blaser MJ. 2004. Bacterial biota in the human distal esophagus. Proc Natl Acad Sci U S A 101:4250-5.

14. Pei Z, Yang L, Peek RM, Jr Levine SM, Pride DT, Blaser MJ. 2005. Bacterial biota in reflux esophagitis and Barrett's esophagus. World J Gastroenterol 11:7277-83.

15. Harris JK, De Groote MA, Sagel SD, Zemanick ET, Kapsner R, Penvari C, Kaess H, Deterding RR, Accurso FJ, Pace NR. 2007. Molecular identification of bacteria in bronchoalveolar lavage fluid from children with cystic fibrosis. Proc Natl Acad Sci U S A 104:20529-33.

16. van der Gast CJ, Walker AW, Stressmann FA, Rogers GB, Scott P, Daniels TW, Carroll MP, Parkhill J, Bruce KD. 2011. Partitioning core and satellite taxa from within cystic fibrosis lung bacterial communities. ISME J 5:780-91.

17. Flanagan JL, Brodie EL, Weng L, Lynch SV, Garcia O, Brown R, Hugenholtz P, DeSantis TZ, Andersen GL, Wiener-Kronish JP, Bristow J. 2007. Loss of bacterial diversity during antibiotic treatment of intubated patients colonized with Pseudomonas aeruginosa. J Clin Microbiol 45:1954-62.

18. Perkins SD, Woeltje KF, Angenent LT. 2010. Endotracheal tube biofilm inoculation of oral flora and subsequent colonization of opportunistic pathogens. Int J Med Microbiol 300:503-11.

19. O'Leary NA, Wright MW, Brister JR, Ciufo S, Haddad D, McVeigh R, Rajput B, Robbertse B, Smith-White B, Ako-Adjei D, Astashyn A, Badretdin A, Bao Y, Blinkova O, Brover V, Chetvernin V, Choi J, Cox E, Ermolaeva O, Farrell CM, Goldfarb T, Gupta T, Haft D, Hatcher E, Hlavina W, Joardar VS, Kodali VK, Li W, Maglott D, Masterson P, McGarvey KM, Murphy MR, O'Neill K, Pujar S, Rangwala SH, Rausch D, Riddick LD, Schoch C, Shkeda A, Storz SS, Sun H, Thibaud-Nissen F, Tolstoy I, Tully RE, Vatsan AR, Wallin C, Webb D, Wu W, Landrum MJ, Kimchi A, et al. 2016. Reference sequence (RefSeq) database at NCBI: current status, taxonomic expansion, and functional annotation. Nucleic Acids Res 44:D733-45.

20. Cole JR, Wang Q, Fish JA, Chai B, McGarrell DM, Sun Y, Brown CT, Porras-Alfaro A, Kuske CR, Tiedje JM. 2014. Ribosomal Database Project: data and tools for high throughput rRNA analysis. Nucleic Acids Res 42:D633-42.

21. Yilmaz P, Parfrey LW, Yarza P, Gerken J, Pruesse E, Quast C, Schweer T, Peplies J, Ludwig W, Glockner FO. 2014. The SILVA and "All-species Living Tree Project (LTP)" taxonomic frameworks. Nucleic Acids Res 42:D643-8.

22. Quast C, Pruesse E, Yilmaz P, Gerken J, Schweer T, Yarza P, Peplies J, Glockner FO. 2013. The SILVA ribosomal RNA gene database project: improved data processing and web-based tools. Nucleic Acids Res 41:D590-6.

23. Katoh K, Asimenos G, Toh H. 2009. Multiple alignment of DNA sequences with MAFFT. Methods Mol Biol 537:39-64.

24. Price MN, Dehal PS, Arkin AP. 2010. FastTree 2--approximately maximum-likelihood trees for large alignments. PLoS One 5:e9490.

25. Kaspar U, Kriegeskorte A, Schubert T, Peters G, Rudack C, Pieper DH, Wos-Oxley M, Becker K. 2016. The culturome of the human nose habitats reveals individual bacterial fingerprint patterns. Environ Microbiol 18:2130-42.

26. Boase S, Foreman A, Cleland E, Tan L, Melton-Kreft R, Pant H, Hu FZ, Ehrlich GD, Wormald PJ. 2013. The microbiome of chronic rhinosinusitis: culture, molecular diagnostics and biofilm detection. BMC Infect Dis 13:210.

27. Tunney MM, Field TR, Moriarty TF, Patrick S, Doering G, Muhlebach MS, Wolfgang MC, Boucher R, Gilpin DF, McDowell A, Elborn JS. 2008. Detection of anaerobic bacteria in high numbers in sputum from patients with cystic fibrosis. Am J Respir Crit Care Med 177:995-1001.

28. Rasmussen TT, Kirkeby LP, Poulsen K, Reinholdt J, Kilian M. 2000. Resident aerobic microbiota of the adult human nasal cavity. Apmis 108:663-75.
